# Combination of Direct Viable Count and Fluorescent In Situ Hybridization (DVC-FISH) as a Potential Method for Identifying Viable *Vibrio* *parahaemolyticus* in Oysters and Mussels

**DOI:** 10.3390/foods10071502

**Published:** 2021-06-29

**Authors:** Jorge García-Hernández, Manuel Hernández, Yolanda Moreno

**Affiliations:** 1 Advanced Center for Food Microbiology, Biotechnology Department, Universitat Politècnica de València, 46022 Valencia, Spain; mhernand@btc.upv.es; 2 Research Institute of Water and Environmental Ingeneering (IIAMA), Universitat Politècnica de València, 46022 Valencia, Spain; ymoren@upv.es

**Keywords:** food safety, *Vibrio parahaemolyticus*, direct viable count, Fluorescence in situ hybridization, DVC-FISH

## Abstract

*Vibrio parahaemolyticus* is a human food-borne pathogen with the ability to enter the food chain. It is able to acquire a viable, non-cultivable state (VBNC), which is not detected by traditional methods. The combination of the direct viable count method and a fluorescent in situ hybridization technique (DVC-FISH) makes it possible to detect microorganisms that can present VBNC forms in complex samples The optimization of the in vitro DVC-FISH technique for *V. parahaemolyticus* was carried out. The selected antibiotic was ciprofloxacin at a concentration of 0.75 μg/mL with an incubation time in DVC broth of 5 h. The DVC-FISH technique and the traditional plate culture were applied to detect and quantify the viable cells of the affected pathogen in artificially contaminated food matrices at different temperatures. The results obtained showed that low temperatures produced an important logarithmic decrease of *V. parahaemolyticus*, while at 22 °C, it proliferated rapidly. The DVC-FISH technique proved to be a useful tool for the detection and quantification of *V. parahaemolyticus* in the two seafood matrices of oysters and mussels. This is the first study in which this technique has been developed to detect viable cells for this microorganism.

## 1. Introduction

Composed of more than 100 species, the *Vibrio* genus is a Gram-negative curved rod bacterium. It is widely distributed in estuarine, marine and freshwater systems worldwide. A large number of Vibrio spp. are in commensal or pathogenic relations with marine organisms such as fish, mollusks and crustaceans [1]. *Vibrio parahaemolyticus*, *Vibrio vulnificus* and *Vibrio cholerae* are the most important human pathogens of this genus [2,3,4,5]. *V. vulnificus* can cause gastroenteritis or severe wound infections due to exposure to highly contaminated water [6,7,8] and, in high-risk groups with their immune systems compromised, it can also cause septicemia [9]. It has been isolated from shellfish, fish, water and sediments of different temperatures and salinities [10]. *V. parahaemolyticus* is recognized as the leading cause of diarrheal disease linked to the consumption of raw, inadequately cooked or contaminated-after-cooking seafood, particularly oysters [6,7,8,11]. The infection with this species is less serious than the one with *Vibrio vulnificus*, and it is generally not associated with death. However, illness is not limited to a particular high-risk group [9]. *Vibrio* spp. occurrence increases to growing areas as water warms up. Therefore, the risk of illness rises during the summer months [12]. In this sense, Garrido-Maestu et al. [3] observed a seasonal distribution of fresh mussels harvested in the southern Rias of Galici having higher densities of *V. parahaemolyticus* in warmer months. Although there are just a few annual cases of infectious diseases related to *Vibrio* spp. in European countries, this number is expected to increase soon due to the consumption of raw seafood, the increase of high-risk patients, the impact of anthropogenic activities and the warming of the marine environment owing to the climate change [8,13,14]. Furthermore, *Vibrio* related outbreaks are more likely to presently occur in geographical areas where these bacteria were once considered absent such as the Pacific Northwest and Northeast coasts of the United States. [15,16,17]. Recently, the Committee for Standardization (CEN) of the European Commission (EC) undertook a work to validate a method designed from the merging of two previous ISO methods for the detection of *V. parahaemolyticus*, *V. cholerae* and other potentially pathogenic vibrio [18]. However, these ISO standard methods did not provide accurate quantification because the VBNC forms were not detected.

The VBNC concept was first discovered in 1982 in *E. coli* and *V. cholerae* environmental samples [19]. Under stress conditions, some microorganisms are capable of entering a viable but non-culturable (VBNC) state as a means of survival, in which they are living cells but unable to grow in their normal environment [20,21]. Thus, it is not possible to detect this state using traditional plating techniques, which lead to the wrong number of the total viable cells in food or environmental and clinical samples due to underestimation [20]. There are many factors that force the VBNC state such as lower nutrient availability and changes in temperature, pH, osmotic pressure and oxygen concentrations [22].

Some authors have demonstrated the VBNC state of *Vibrio* spp. by inducing stress–response genes as well as genes involved in the regulation of cold protection [21,23,24]. Moreover, Nowakowska and Oliver [25] described the ability of *V. vulnificus* cells to enter into a VBNC state when water temperatures were below 13 °C. *V. parahaemolyticus* is sometimes found entering the VBNC state in unfavorable conditions such as low temperature in aquatic environments [26]. It can still express some metabolic activity, so it can continue to cause infectious diseases in humans [27,28].

According to the recent ISO 21872-1:2017, which describes a method for the determination of potentially enteropathogenic *V. parahaemolyticus*, *V. cholerae* and *V. vulnificus*, the use of traditional culture techniques for detecting *V. parahaemolyticus* may require about 4–5 days. The methodology consists of four steps: a primary enrichment, a secondary enrichment, isolation in selective culture media and a final confirmation by biochemical assays or PCR. Molecular techniques such as fluorescent in situ hybridization (FISH) and PCR can be used as alternatives to conventional detection methods, although they do not allow differentiation between non-viable and viable cells [29], which can result in false positives [30]. Fluorescent in situ hybridization (FISH) is a method that allows the detection and identification of microbial organisms (bacteria, protozoa and yeasts) at the species or genus level using fluorescence-labeled, rRNA-targeted oligonucleotide probes, followed by an analysis with a fluorescence microscope [31,32].

The direct viable count (DVC) procedure involves exposing bacterial cells to a revival medium that contains antibiotics that prevent cellular division [33]; elongated cells are then enumerated as viable cells [34]. DVC is useful for the detection of specific bacterial viable cells in combination with a microscopic technique such as FISH [31]. DVC assay is based on the incubation of bacterial cells in a medium containing antibiotics that inhibit DNA gyrase and prevent cell division. The resulting cells can continue to metabolize nutrients and elongate and/or increase their size during incubation [31,35]. These viable cells are then counted and distinguished from non-viable cells by size [36]. The combination of the DVC procedure, which increases intracellular rRNA levels, with FISH performed on rRNA-targeted sequences, could prove useful in detecting and identifying viable cells in mixed microbial communities [37]. Armisen and Servais (2003) [38] combined for the first time direct viable count (DVC) and fluorescent in situ hybridization (FISH) to enumerate viable E-coli in rivers and wastewaters. A combination of the DVC procedure and FISH facilitates the detection and identification of viable cells in mixed microbial populations [39]. Several antibiotic inhibitors of DNA gyrase have been used in previous trials of DVC-FISH with other microorganisms [40,41]. Ciprofloxacin is a fluoroquinolone that has been shown to be a good inhibitor of DNA gyrase for both Gram-positive and Gram-negative bacteria [42].

The aim of this work was to optimize DVC-FISH as a rapid, sensitive and specific method for *V. parahaemolyticus* detection, particularly in bivalves such as oysters and mussels. In addition, some molecular techniques were compared to determine the viability and evolution of such pathogens in bivalve samples submitted to different temperatures.

## 2. Materials and Methods

### 2.1. Bacterial Strains and Culture Conditions

The *Vibrio parahaemolyticus* strain CECT511 (Spanish Type Culture Collection, Valencia, Spain) was used for all assays. This bacterium was grown in TSA (Trypic Soy Agar, Merck, Darmstadt, Germany) modified with 2% NaCl (mTSA), and it was shown to be more efficient to recover *V. parahaemolyticus* than inTSA agar [43]. Plates were incubated at 37 °C for 24 h in aerobic conditions as CECT-recommended.

### 2.2. DVC Assays

Experimental assays were carried out to determine the optimal conditions of the DVC technique. An exponential phase culture (16 h) of *V. parahaemolyticus* was inoculated in TSB medium (Trypic Soy Broth, Merck, Darmstadt, Germany) with 2% NaCl (mTSB) at four different ciprofloxacin concentrations (0.5, 0.75, 1 and 1.5 μg/mL). They were then incubated for different times (5, 6, 7, 8 and 16 hours) at 37 °C. Following that, 1 mL aliquots were fixed for subsequent analysis by FISH, which is described below. A count of the initial inoculum was made by serial decimal dilutions in 9 mL of PBS 1X (phosphate-buffered saline), of which 100 μL were plated in mTSA plates with 2% of NaCl and incubated at 37 °C for 24 h. A control tube of mTSB broth containing no antibiotic was inoculated with *V. parahaemolyticus*. Thus, the original cellular size of *V. parahaemolyticus* was determined by FISH. All assays were performed in duplicate.

### 2.3. Fluorescent In Situ Hybridization (FISH) Analysis

FISH analysis was performed using the *V. parahaemolyticus* 16S rRNA oligonucleotide-specific probe (VP612: 5′-TGCAATTCCGAGGTTGAGCCCCGG-3′) [44,45]. This probe was labelled with CY3 fluorophore. The EUB338 universal probe mixture, complementary to a region of the 16S rRNA of the domain bacteria [44] was used as a positive control. A mixture of three EUB338 universal probes, complementary to a 16S rRNA region of eubacteria, was used as a positive control [46]. These probes were labeled with 5 (6)-carboxyfluorescein-N-hydroxysuccinimide ester (FLUOS).

For FISH analysis, 1 mL of DVC broth was centrifuged (8000× *g* for 8 min). The resulting pellet was resuspended in 1 mL PBS 1X buffer to wash the cells and then centrifuged (8000 rpm for 8 min). Samples were fixed with paraformaldehyde (PFA) 4%, and 10 μL of fixed sample was placed on a gelatin-coated slide, air dried, dehydrated (50, 80, 100% ethanol) and hybridized with VP612 and EUB338 16S rRNA probes [46]. To provide a specific hybridization, a final concentration of 30% formamide was included in the hybridization buffer.

Hybridized samples were visualized with a fluorescence Olympus microscope BX50 equipped with U-MWB, U-MWIB and U-MWIG exciter filters. Color micrographs were taken with an Olympus DP-10 digital camera (Olympus Optical Co., Hamburg, Germany). Cells that were elongated to at least twice their original size compared with the exponential culture control were estimated to be viable cells, as described by other authors [40,47]. Bacterial size elongation was evaluated by an ocular micrometer.

### 2.4. DVC-FISH Sensitivity

A *V. parahaemolyticus* overnight culture grown in mTSA at 37 °C for 24 h was 10-fold serially diluted in PBS buffer to obtain cultures between 1 and 10^7^ CFU/mL. An aliquot of 1 mL of each dilution was inoculated in DVC medium (mTSB with 2% NaCl and 0.75 μg/mL of ciprofloxacin) and incubated at 37 °C. After 5 h of incubation, 1 mL of each flask medium was taken, and the cells were fixed and processed for in situ hybridization as described above. Counts of viable cells were determined as the mean value obtained from 20 microscopic fields from two different slides. We estimated viable cells as those which were elongated to at least twice their original size. The number of viable cells/mL were obtained by multiplying the average of the counts by the microscope factor and the dilution applied. The initial concentration of each dilution was determined by a duplicate plate count in TSA medium with 2% NaCl.

### 2.5. Detection of Viable V. parahaemolyticus in Inoculated Samples

Oysters and mussels were bought in the Central Market of Valencia, Spain. The weight of the oyster and mussel meat was between 7 and 11 g for oysters and between 12 and 16 g for mussels. The meat was extracted and treated under UV light (260 nm) for 45 min. The absence of *Vibrio* spp. cells was verified by culture on TCBS medium (agar-thiosulfate-citrate-bile salts-saccharose) and mTSA. It was also verified by FISH analysis that there was no presence of *Vibrio parahaemolyticus* cells. Briefly, a portion of the sample was homogenized in PBS buffer (1:10) using a Stomacker^®^ homogenizer (VWR, Barcelona, Spain), and subsequently, 100 μL were spread on the plates and incubated at 37 °C for 24 h in aerobic conditions. FISH analysis was performed as described in Section 2.3.

To determine the usefulness of the DVC-FISH method in *Vibrio* survival studies, an assay was performed in order to simulate contaminated samples from their purchase in the supermarket until their refrigeration at home. Three replications of this experiment were performed, and the results shown are an average of these. Firstly, the transport from supermarket to home was simulated at 20 °C. In short, 5 mL of an overnight culture of *V. parahaemolyticus* was added to 5 g of oysters and mussels both treated under UV, respectively, and incubated at 20 °C. At 0, 10 and 20 min, volumes of 1 mL of the inoculated samples were introduced in different tubes containing 9 mL of PBS 1X. Product refrigeration carried out at home under different conditions was also imitated (4 °C and 8 °C). For this purpose, 1 mL of an overnight culture of *V. parahaemolyticus* was added to 12 tubes, each containing 1 g of oysters and mussels treated under UV. Six of them were incubated at 4 °C, and the other 6 at 8 °C. At 0, 1, 5, 24, 48 and 96 h of incubation, the entire contents of a tube from each temperature were introduced into different flasks containing 9 mL of PBS 1X. Finally, 4 mL were taken from each PBS suspension: 1 mL for FISH analysis, 1 mL for analysis by DVC-FISH, 1 mL for DNA extraction and 1 mL was destined to serial decimal dilution in physiological solution to determine the CFU/mL (colony-forming units). 100 μL of each decimal dilution were plated on mTSA (NaCl 2%) medium supplemented with 0.1% sodium pyruvate for the resuscitation of viable but non-culturable *Vibrio parahaemolyticus* cells with conditions induced at low temperatures [48]. Plates were incubated under optimal conditions as described above.

### 2.6. Viability Staining

To determinate live and dead cells, 500 μL of each *V. parahaemolyticus* suspension were inoculated with 0.8 μL of LIVE/DEAD^®^ BacLight Kit (Molecular Probes, Eugene, OR, USA) and incubated at room temperature in darkness under agitation for 5–10 min following the manufacturer´s recommendations. An aliquot of 10 μL was placed on a diagnostic slide coated with poly-L-lysine (0.01%) (Sigma), and live and dead cells were counted under an epifluorescence microscope using a double band-filter cube (XF 53, Omega, Brattleboro, USA).

### 2.7. Nucleic Acid Extraction

The stability of the RNA and DNA was also studied as a viability marker. Inoculated samples of oysters and mussels were immediately processed for RNA extraction. They were centrifuged at 14,000× *g* for 5 min, the supernatant was removed and RNA was extracted using a commercial kit (Hight Pure RNA Isolation Kit, Roche, Madrid, Spain), following the manufacturer’s directions. Next, the RNA was reverse-transcribed to cDNA, using a commercial reaction kit (Transcriptor First Strand cDNA Synthesis Kit, Roche) by following the manufacturer’s protocol. For DNA isolation, aliquots were also centrifuged at 14,000× *g* for 5 min, the supernatant was removed and DNA was extracted by the commercial system “Real Pure Spin Kit” (REAL™, Durviz, Spain). DNA was stored at −20 °C until PCR was done.

### 2.8. Detection and Testing of Nucleic Acid Stability of V. parahaemolyticus

A PCR was carried out to detect and check the RNA and DNA stability in *V. parahaemolyticus* cells, using L-tl and R-tl primers [49], which amplified a specific, 450 pb fragment of the *tl* gene sequence (located between nucleotides 781 and 1230) that encoded the thermolabile hemolysin. Sensitivity and specificity of the primers, as well as the amplification cycle conditions, were tested in a previous study [49].

## 3. Results

### 3.1. DVC-FISH Assay Optimization

**Figure 1:**
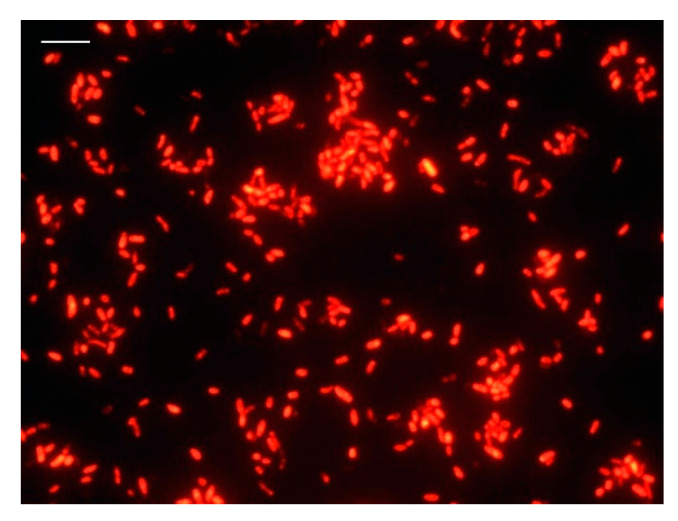
FISH micrographs showing the hybridization of *V. parahaemolyticus* cells, showing the usual cell size as the control. (bar: 10 μm).

An optimized protocol was established for the specific detection of viable *V. parahaemolyticus* cells. Figure 1 shows the control of the original size of the *V. parahaemolyticus* cells without incubation with DVC broth.

Four concentrations of ciprofloxacin (0.5, 0.75, 1 and 1.5 μg/mL) and five incubation times (5, 6, 7, 8 and 16 h) were tested.

No cells were observed after 16 h of incubation at any concentration.

A concentration of 0.5 μg/mL did not produce enough cell elongation at any of the incubation times. Although the cells were elongated after 7 and 8 h of incubation, at this concentration, they did not increase their size to more than twice the original.

**Figure 2:**
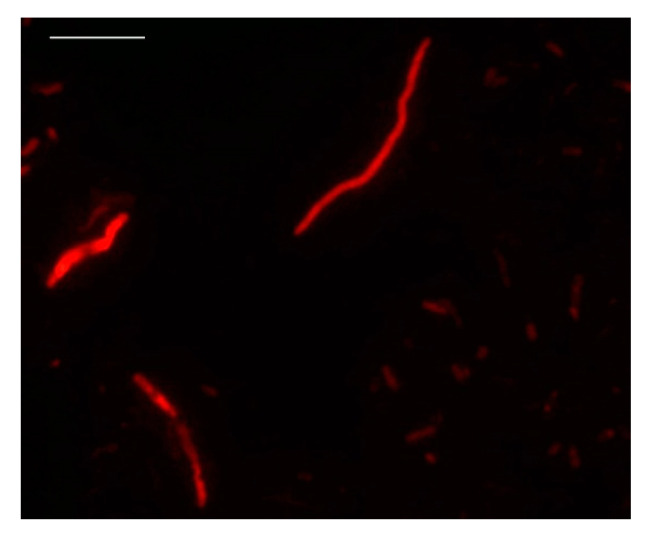
Viable but degraded *V. parahaemolyticus* cells after incubation in DVC broth (0.75 μg/mL—7 h) (bar: 10 μm).

After 7 or more hours of incubation and a high ciprofloxacin concentration (0.75, 1 and 1.5 μg/mL), cells appeared degraded in all cases (Figure 2).

In the subsequent assays, the concentrations of 0.75, 1 and 1.5 μg/mL of ciprofloxacin were tested after 5 and 6 h of incubation. Elongation of viable cells occurred with the three concentrations of ciprofloxacin (0.75, 1 and 1.5 μg/mL), although at 0.75 μg/mL, greater elongation was detected. Moreover, a drop in the number of cells was observed by LIVE/DEAD fluorescent assay at concentrations of 1 and 1.5 μg/mL. In addition, 0.75 μg/mL and 5 and 6 h of incubation yielded similar results, as it was possible to observe viable elongated cells without any alteration associated with the presence of an antibiotic.

**Figure 3:**
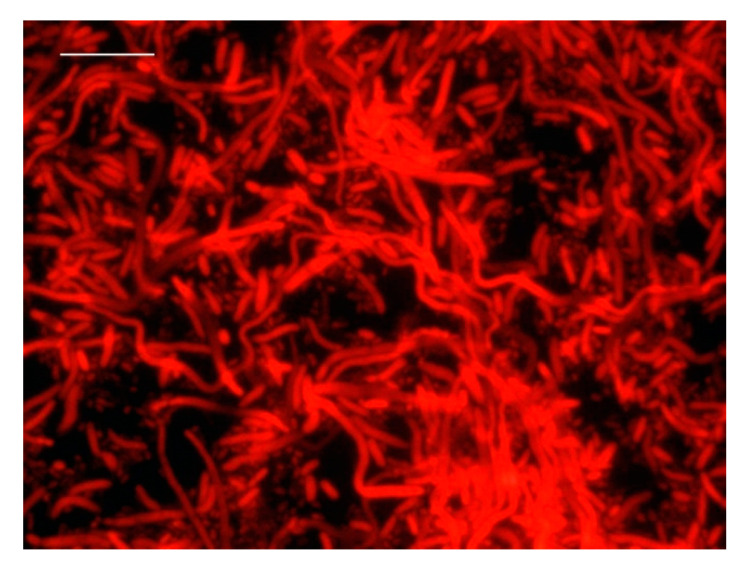
Detection by FISH of live and dead cells of *V. parahaemolyticus* after 5 h incubation with ciprofloxacin (0.75 μg/mL). (bar: 10 μm).

Results showed that long incubation periods in the presence of an antibiotic caused cell degradation. Therefore, the optimal conditions for DVC analysis turned out to be 0.75 μg/mL of ciprofloxacin and 5 h of incubation (Figure 3).

Once the optimal conditions for the DVC procedure were selected for *V. parahaemolyticus,* the number of viable cells before and after the DVC incubation was also tested by both culture on mTSA as described in Materials and Methods and by using the LIVE/DEAD BacLight kit. A DVC broth without an antibiotic, inoculated with the same *V. parahaemolyticus* inoculum and incubated for 5 h, was used as the control. As shown in Table 1, the results obtained by culture were similar to those obtained by DVC-FISH assay, meaning that that almost all cells were both viable and cultivable for these conditions. Furthermore, in the DVC medium, the number of cells was not affected by 5 h of incubation, and the antibiotic did not seem to affect bacteria viability. Therefore, the selected antibiotic concentration and incubation time results were optimal, taking into consideration that cell elongation was achieved without degradation or replication of the cells. It was observed that in the control culture (DVC broth without antibiotic), viable cells were not elongated, and cells counts were higher than the ones obtained at time 0 h due to their ability to replicate.

**Figure 4:**
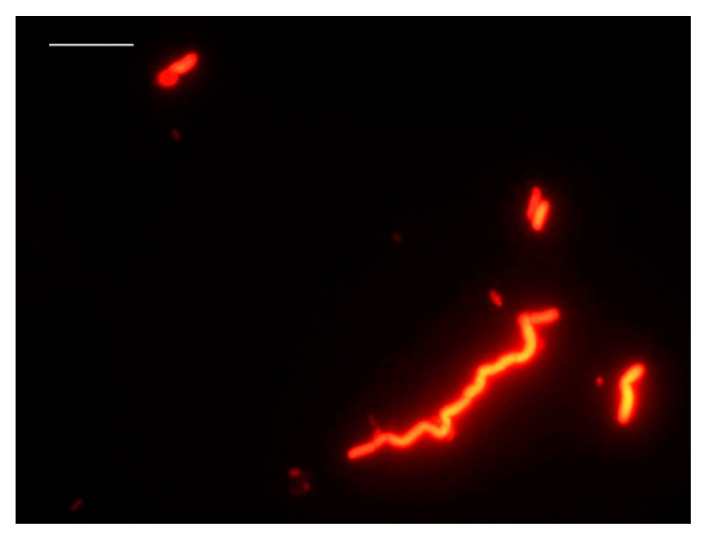
Detection of viable *V. parahaemolyticus* cells by DVC-FISH at a concentration of 10^2^ cells/mL. (bar: 10 μm).

Starting from an initial culture of 2.5 × 10^8^ cfu/mL of *V. parahaemolyticus,* serial decimal dilutions were made in order to determine the detection limit of the DVC-FISH technique. According to the presence and appearance of the cells in each dilution, the detection limit was established at 10^2^ cells/mL (Figure 4).

### 3.2. Detection of Viable V. parahaemolyticus in Inoculated Samples

The DVC-FISH technique was applied in real samples inoculated with *V. parahaemolyticus* to assess its usefulness in detecting viable cells of the pathogen in two bivalve mollusks, oysters and mussels. The results were compared with the ones obtained when applying the culture to mTSA agar plates to detect viable bacteria. Different conditions were simulated to ascertain the growth kinetics of *V. parahaemolyticus* in contaminated oysters and mussels until their arrival to the consumer. The process called “transport”, defined as the time passed from the purchase of a food until its storage in the refrigerator, was simulated for oysters and mussels at 22 °C for 10, 20 and 30 min. As shown in Table 2, the initial inoculum of *V. parahaemolyticus* in oysters and mussels contained 1.7 × 10^7^ cfu/mL and 1.3 × 10^6^ cfu/mL, respectively. The bacterial plate counts increased in both samples throughout the time. The same tendency was detected in the FISH and DVC-FISH counts, which were slightly higher than those obtained by culture. FISH counts were the highest due to the count of VBNC, viable and non-viable cells.

Subsequently, the microbiological processes that occur when food is stored in refrigeration were simulated at 4 and 8 °C for different periods of time. In the simulation at 4 °C, the initial inocula for the samples of oysters and mussels were around 1.8 × 10^7^ cfu/mL, respectively (Table 3). Results showed that the concentration of the culture and the total cells went down progressively. Viable cells were identified by the DVC-FISH technique throughout the whole refrigeration process at 4 °C (96 h). The counts obtained by cultivation and FISH decreased by between three and five orders of magnitude for both oysters and mussels. However, after 48 h in oysters and 96 h in mussels, *V. parahaemolyticus* was not detected by culture, even though the cells were elongated after the DVC. For the simulation at 8 °C, the oysters’ and mussels’ initial inocula were around 1.8 × 10^7^ cfu/mL, respectively (Table 4). A reduction was also reported, but more gradually than at 4 °C. A decrease of 3–4 orders of magnitude was observed by traditional cultivation. DVC yielded counts which were one log unit higher than the culture counts. FISH analysis, as resulting from the simulation at 4 °C, generated the highest counts.

### 3.3. Detection and Testing of NA Stability of V. parahaemolyticus

Bacterial DNA and RNA were isolated from all the samples of oysters and mussels. PCR and later, electrophoresis, were performed to verify the stability of the *V. parahaemolyticus* nucleic acids. The specific amplifications for the *tlh* gene from both the DNA and RNA of *V. parahaemolyticus* were detected in all the assays and times analyzed. Therefore, although the temperature affected the growth of the culture, DNA and RNA remained stable without degrading.

## 4. Discussion

In this study, a combined DVC-FISH method was optimized for the specific detection and quantification of viable pathogenic *V. parahaemolyticus* in two bivalve mollusks, oysters and mussels. The developed method reduced the total detection and identification time of *V. parahaemolyticus* in these seafoods by up to 4 h, which could be a good complement to detection by traditional cultivation methods such as the official ISO 21872-1:2017 method, which requires a total analysis time of 4 days.

The FISH method is a useful tool for the initial identification of microbial pathogens, and it is considered quicker and associated with less hands-on time than traditional culture methods [32,50]. However, this technique on its own cannot ensure that bacteria remain alive.

In this regard, the DVC technique has been widely applied to the study of aquatic samples, including seawater [51,52], groundwater [53], wastewater [54], fresh water [36,37,55] and drinking water [39,56,57]. It has also been used for the study of cell viability in food samples [58]. In this method, bacteria are exposed to a medium that contains a sublethal concentration of an antibiotic substance, which prevents cell division by inhibiting DNA gyrase without affecting other metabolic activities such that cell growth is not impeded [35]. Therefore, when cells are cultivated under these conditions, the amount of ribosomal RNA in active cells increases, resulting in elongated cells [35], which are then enumerated as viable cells because dead cells remain unchanged [59]. Cells are considered viable when their length is at least twice the original one [58]. It is essential that no cell division occurs in order to ensure that the DVC analysis is accurate, which is generally confirmed by comparing cell presence before and after incubation [52]. However, in the DVC medium, bacterial cell division can be underestimated due, among other factors, to cell lysis caused by high concentrations of antibiotics, which can counterbalance the increase in the number of bacteria because of cell division. On the other hand, cell division can be overestimated in some strains that are resistant to antibiotics when there is an insignificant increase in their cell number compared with the total bacteria count but that is significant for their own population [31]. Therefore, in order to obtain reliable and accurate results, it is fundamental to select the antibiotic and its dose properly.

Due to the lack of success with some culture methods [60], and as PCR only indicates the presence of DNA, a combination of molecular techniques such as DVC-FISH seems to be the most efficient strategy for the detection and the quantification of viable cells, including those in a VBNC state. In fact, it has been reported regarding the detection of viable cells of *E. coli* in various types of samples [61], *H. pylori* in wastewater and drinking water [62], *L. monocytogenes* in complex mixed communities such as wastewater samples [30], *L. delbrueckii* subsp. *bulgaricus* and *S. thermophilus* in inoculated feces samples [36] and *Yersinia* spp. in minced pork meat [50].

To ensure the inhibition of cell division and the maximum elongation of bacteria in aquatic samples, different combinations of several antibiotics (mainly nalidixic, piromidic and pipemidic acid as well as ciprofloxacin) have been proposed [42,51,53,57]. It has been demonstrated in many studies that *V. parahaemolyticus* exhibit multiple-antibiotic resistance due to the misuse of antibiotics to control infections in aquaculture production [63]. In a research on antimicrobial resistance, Yano et al. [64] revealed the occurrence of resistance to nalidixic acid and ampicillin in *V. parahaemolyticus* strains derived from shrimp. The selected antibiotic for the current research, ciprofloxacin, is an effective inhibitor of cell division in Gram-positive and Gram-negative bacteria, and its effectiveness has been proven in *V. parahaemolyticus* strains derived from water and marine samples [65,66] and from oysters [67]. The DVC medium with 0.75 μg/mL of ciprofloxacin turned out to be effective in preventing cell division of *V. parahaemolyticus* and promoting the elongation of viable cells after 5 h of incubation. Once the DVC-FISH procedure was optimized, the detection limit of the technique was established at 10^2^ cells/mL in inoculated samples. This limit could be improved by centrifuging the whole amount of DVC broth instead of just 1 mL.

However, the DVC-FISH technique has certain limitations. Thus, as indicated by Huber et al. [68], despite the high specificity of FISH and the possibility of direct quantitative imaging, some of its key constraints are that the time of the assay and the probe consumption prevent its regular use in diagnostics. The influence of the fixation/permeabilization step [69] and the design of specific probes which need to be specific, sensitive, and relatively short-chained to make cell penetration easier [70] have also been pointed out. The selection of the appropriate DNA gyrase-inhibitor antibiotic, its concentration and the necessary incubation time are also factors that must be taken into account. Finally, background noise in the sample sometimes limits the ability to observe and determine cell size (36, 41, 58).

In order to determine the suitability of the optimized technique to detect viable cells of *V. parahaemolyticus* in seafood samples, DVC-FISH was applied to track the pathogen viability through a survival study. *Vibrio parahaemolyticus* is a Gram-negative bacteria related to seafood-associated illness, which not only causes symptoms of gastroenteritis, but also wound infections and septicemia [71]. Numerous cases of gastroenteritis caused by *V. parahaemolyticus* have been detected and isolated in Spain, Greece, Britain, Denmark and other countries in Europe over the years [72,73]. Love et al. [74] demonstrated that the cold chain was effective in reducing the risk of *V. parahaemolyticus* in seafood by modeling the effects of temperature on the growth of this bacterium in oysters. Moderate bacterial growth was detected in oysters that had not entered the cold chain, while bacterial growth stopped once they were under refrigeration. This information is in line with the results of the present study, where it was observed that both bacterial counts and viability of *V. parahaemolyticus* in samples of oysters and mussels increased throughout the time of transport at 20 °C, from the purchase to the storage of the food in the refrigerator. However, a rapid decrease in viable and, therefore, potentially virulent cells was detected when the samples were under refrigeration at 4 °C and 8 °C. The same trend was detected in the study carried out by López-Joven [75], where the bacterial load of *V. parahaemolyticus* in Manila clam samples was lower at 4 °C and 15 °C than at 28 °C in all treatments. Therefore, the control of temperatures and times in the production and transport chains as well as optimal manipulation, preparation and storage conditions are crucial to ensure food safety. The risk of *Vibrio* spp. can be attenuated with shorter times of storage and the rapid cooling of seafood [76]. The wrong storage temperature at above 20 °C, even with short storage times, could increase the risk of *V. parahaemolyticus* infection through contaminated seafood if the necessary bacterial load was reached because cells of this pathogen remain metabolically active at this temperature, as reported in our work.

Our results showed that a higher number of samples were recorded as positive for *V. parahaemolyticus* by DVC-FISH analysis than by culture, since the first included the VBNC cells. Furthermore, this technique allowed us to specifically identify and quantify the viable cells directly in a few hours. Similar results have been reported for the detection and enumeration of other pathogens such as *L. monocytogenes* in wastewater samples [30], where the DVC-FISH technique was more sensitive than PCR and culture methods. Furthermore, an advantage of this technique is that in samples with an initial contamination of the pathogen above 10^2^ viable cells/g, prior culture enrichment is not required due to the detection limit obtained. mTSA medium was also applied to compare the results with the ones obtained by molecular techniques. The presence of V. *parahaemolyticus* has been commonly detected by different culture-based methods [77]. However, our results revealed that traditional culture methods can be limited in detecting *V. parahaemolyticus* in food matrices because the cells can acquire a VBNC state, in which they are metabolically active but unable to grow on a synthetic media [77].

As demonstrated in this study, DNA remains stable under stress conditions that cause cell death, so PCR is not a useful technique because it is unable to distinguish between viable and non-viable cells in food samples [29], which leads to false positive results [78]. Moreover, RNA was detected with the same intensity throughout the survival assays. Hence, in this case, the expression of the *tlh* gene was not a good viability marker to detect viable *V. parahaemolyticus*. Other authors reported the inefficiency of RNA detection as bacterial viability identification [79].

The combination of traditional culture methods with the DVC-FISH could turn into the most efficient strategy for the detection and quantification of *V. parahaemolyticus* in bivalve matrices. Our results show that the DVC–FISH method is a useful and fast tool for the identification and quantification of viable forms of *V. parahaemolyticus* in oysters and mussels and could be used for both survival studies and food quality control in naturally contaminated samples.

## 5. Conclusions

The DVC–FISH method is a useful and fast tool for the detection and quantification of viable forms of *V. parahaemolyticus* in oysters and mussels and could be used for both survival studies and food control. The optimal conditions were 0.75 μg/mL of ciprofloxacin in DVC broth and 5 h of incubation. The developed method allowed us to detect and identify viable *V. parahaemolyticus* in inoculated seafood in just 4 h, which was an excellent complement to the ISO standard, which requires a time of several days.

In the process of simulating transport conditions of mussels and oysters at 22 °C, bacterial counts by culture, FISH and DVC-FISH increased significantly over time. However, the viable cell counts obtained by DVC-FISH were more accurate than the ones obtained by culture because both cultivable and VBNC cells were detected by this technique. In the simulation process of refrigeration conditions at 4 and 8 °C for oysters and mussels, *V. parahaemolyticus* counts decreased by between three and four orders of magnitude by cultivation and by FISH. After 48 h in refrigeration, not only did *Vibrio parahaemolyticus* lose the ability to grow, but the cells also did not elongate after DVC. Finally, the *V. parahaemolyticus* nucleic acid stability test through specific amplification of the *tlh* gene determined that the DNA and RNA remained stable, without degrading during the process.

## Figures and Tables

**Table 1:** Results of culture viability using the LIVE/DEAD BacLight kit.

Time	Viable (SYTO) (cells/mL)	Not Viable (PI) (cells/mL)	mTSA Plate Count (cfu/mL)	FISH (cells/mL)
Initial culture (0 h)	9.6 × 10^6^	3.4 × 10^6^	1.4 × 10^7^	1.5 × 10^7^
DVC (5 h)	2.4 × 10^7^	3.3 × 10^7^	1.7 × 10^7^	3.2 × 10^7^
Control (5 h)	6.6 × 10^7^	2.6 × 10^7^	7.3 × 10^7^	1.3 × 10^8^

**Table 2:** Counts of oyster and mussel samples in transport simulation.

Time (Minutes)	mTSA Plate Count (cfu/mL)		FISH (cells/mL)		DVC-FISH (cells/mL)	
	Oysters	Mussels	Oysters	Mussels	Oysters	Mussels
0	1.70 × 10^7^	1.30 × 10^6^	1.80 × 10^7^	9.70 × 10^6^	1.70 × 10^7^	4.90 × 10^6^
10	7.00 × 106	6.90 × 10^6^	8.70 × 10^6^	2.10 × 10^7^	8.10 × 10^6^	9.70 × 10^6^
20	8.80 × 106	9.70 × 10^6^	1.40 × 10^7^	4.00 × 10^7^	9.40 × 10^6^	2.80 × 10^7^

**Table 3:** Counts of oyster and mussel samples in refrigeration simulation at 4 °C.

Time (Hours)	mTSA Plate Count (cfu/mL)		FISH (cells/mL)		DVC-FISH (cells/mL)	
	Oysters	Mussels	Oysters	Mussels	Oysters	Mussels
0	9.50 × 10^6^	1.65 × 10^7^	1.80 × 10^7^	1.97 × 10^8^	7.04 × 10^6^	2.23 × 10^7^
1	6.24 × 10^6^	1.02 × 10^7^	6.09 × 10^6^	1.57 × 10^8^	3.93 × 10^6^	1.74 × 10^7^
5	2.00 × 10^5^	8.10 × 10^5^	1.78 × 10^6^	8.54 × 10^7^	1.36 × 10^6^	8.54 × 10^6^
24	1.57 × 10^4^	2.06 × 10^5^	8.80 × 10^5^	9.76 × 10^6^	1.40 × 10^5^	1.44 × 10^6^
48	<10	4.50 × 10^3^	1.68 × 10^4^	3.50 × 10^5^	6.40 × 10^3^	7.80 × 10^4^
96	<10	<10	1.30 × 10^3^	6.50 × 10^4^	6.20 × 10^2^	7.30 × 10^3^

**Table 4:** Counts of oyster and mussel samples in refrigeration simulation at 8 °C.

Time (Hours)	mTSA Plate Count (cfu/mL)		FISH (cells/mL)		DVC-FISH (cells/mL)	
	Oysters	Mussels	Oysters	Mussels	Oysters	Mussels
0	1.65 × 10^8^	1.78 × 10^7^	1.90 × 10^8^	7.80 × 10^8^	2.00 × 10^8^	1.83 × 10^7^
1	1.03 × 10^8^	1.50 × 10^6^	1.41 × 10^8^	7.19 × 10^7^	1.61 × 10^8^	7.32 × 10^6^
5	8.50 × 10^6^	1.70 × 10^5^	4.80 × 10^7^	6.34 × 10^7^	1.31 × 10^7^	1.44 × 10^6^
24	2.26 × 10^6^	7.70 × 10^4^	6.30 × 10^6^	3.53 × 10^7^	2.40 × 10^6^	1.22 × 10^6^
48	3.50 × 10^5^	4.70 × 10^4^	1.30 × 10^6^	7.32 × 10^6^	8.30 × 10^5^	1.30 × 10^5^
96	1.98 × 10^4^	1.10 × 10^4^	2.11 × 10^5^	1.22 × 10^6^	7.60 × 10^4^	8.20 × 10^4^
